# Design and Implementation of Ultrasonic Impact Peening-Based Device for Stainless Steel Surface Texture Fabrication

**DOI:** 10.3390/mi12070787

**Published:** 2021-06-30

**Authors:** Wangjie Hu, Qiang Zhang, Xiaohui Wang, Dongxu Zhao, Zhenjiang Hu, Xuesen Zhao, Haiyan Shao, Xiuhua Men, Tao Sun

**Affiliations:** 1 Center for Precision Engineering, Harbin Institute of Technology, Harbin 150001, China; mattijj0424@foxmail.com (W.H.); qiangzi0814@163.com (Q.Z.); zdx808@163.com (D.Z.); lyhoo@hit.edu.cn (Z.H.); spm@hit.edu.cn (T.S.); 2 School of Mechanical Engineering, University of Jinan, Jinan 250022, China; me_shaohy@ujn.edu.cn (H.S.); me_menxh@ujn.edu.cn (X.M.)

**Keywords:** stainless steel, surface texture fabrication, ultrasonic impact peening, device design

## Abstract

The manufacturing of precise surface microstructures with low cost is needed for surface texturing-based surface engineering. In this paper, a device for the fabrication of surface microgroove texture on stainless steel based on ultrasonic impact peening (UIP) is proposed and investigated. First, the principle of applying the UIP into the fabrication of surface texture is analytically described. Then, the design of the UIP device, particularly the design of functional systems and mechanical structures, is carried out. Next, a UIP experimental device is built, and is further applied to fabricate microgroove textures on 316L stainless steel. The subsequent experimental characterization of microgroove morphology demonstrates the feasibility of the designed UIP device for the fabrication of stainless steel surface texture.

## 1. Introduction

Stainless steel has been widely used in various engineering fields due to its high corrosion resistance, high tensile strength, and easy formability. However, the poor wear resistance of stainless steel greatly restricts its performance [1]. In recent decades, considerable work reported that the friction and wear performance of stainless steel surfaces can be tailored by introducing surface textures [2,3,4,5,6,7,8,9]. However, how to fabricate high-precision surface textures on ferrous metal of stainless steel with low cost and high efficiency is one of the key problems. 

The methods of fabricating surface textures include mechanical processing [10], energy beam processing [11], chemical etching [12], and so on. In particular, laser surface texturing (LST) has been widely used in texture fabrication for its wide range of applicable materials, high precision and high efficiency [6,8]. Cui et al. [13] used a nanosecond laser to fabricate array textures with a height of 50−100 μm on 17-4PH stainless steel. They found that the texture presents obvious thermal damage under the action of laser ablation, leading to irregular morphology and rough surface. Wang et al. [14] used a femtosecond laser to fabricate array microgrooves with a depth of 12 μm on 304L stainless steel. Since the time scale of femtosecond laser ablation is very short, the small heat-affected zone results in strong improvement in the shape accuracy compared with nanosecond laser. However, the quality of the textured surface is still not high due to the inevitable thermal effect of laser ablation. High-energy laser irradiation can easily induce metal surface damage and metallographic changes, which subsequently affect the physical and mechanical properties of ablated materials. In addition, it is also challenging for LST to fabricate shallow textures in sub-micron range of feature size while maintaining high precision, and the cost of related equipment is relatively high [15,16,17,18,19]. Recently, ultra-precision diamond machining methods have also been proposed to fabricate high-precision surface textures [20,21,22,23,24]. In particular, due to the significant thermal diffusion between carbon and iron, this may cause severe thermochemical tool wear, therefore reducing machining accuracy. Zhang et al. [25] fabricated square and sinusoidal surface textures with a minimum depth of 1.3 μm and a period of 90 μm on SUS420J2 stainless steel inevitable by elliptical vibration cutting (EVC) technology. The prepared texture shows good precision and consistency. However, this technology needs to be performed on ultra-precision machine tool in conjunction with specific elliptical vibration equipment, which places extremely high requirements on equipment and technology. Therefore, a low-cost and high-precision technique for the fabrication of stainless steel surface texture is highly desired.

Ultrasonic impact peening (UIP) is a widely used surface treatment method to eliminate the surface residual tensile stress of welded metallic part and improve its fatigue life [26,27,28,29,30]. Malaki et al. [31] explained the effect of UIP on the microstructure, hardness, fatigue strength, and life cycle of stainless steel part. They found that the UIP-based surface treatment leads to the formation of nanocrystalline layer, which greatly contributes to the improvement of hardness, fatigue strength, and life cycle. The basic principle of UIP technology is as follows: the ultrasonic impact tool continuously impacts the workpiece surface at ultrasonic frequency, which causes the surface to produce great compression-plastic deformation, accompanied with the refinement of surface grains and surface strengthening [32,33,34]. Be different from the ultra-shallow processing depth when the UIP is traditionally used for surface treatment, the processing depth is greater when the UIP is applied to the fabrication of textures. Based on UIP, by controlling its processing precision and trajectory, it is expected to realize the fabrication of textures on stainless steel surface. UIP technology has been developed for many years, and related technologies and products have been very mature. The price of UIP devices is much lower than that of ultra-precision machining tools. Although expensive diamond tools are needed in diamond cutting, low-cost carbide tools can be used as the tool material in UIP processing. Thus, UIP technology is expected to become a low-cost and high-precision fabrication technology for surface textures, in turn broadening the practical industrial application of stainless steel and the application scope of UIP technology itself. However, there is no report on the fabrication of stainless steel surface texture using UIP. Therefore, in this work we design a UIP device and explore the feasibility of applying UIP method in the fabrication of surface texture on stainless steel.

## 2. Principle of Surface Texture Fabrication Based on UIP

**Figure 1:**
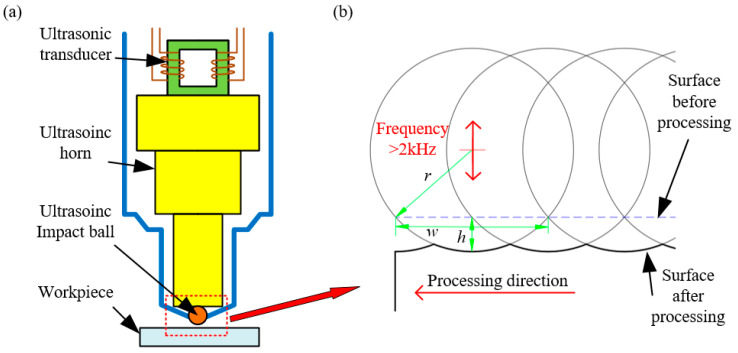
Principle of surface texture fabrication based on UIP. (**a**) Global view; (**b**) Enlarged view.

Principle of stainless steel surface texture fabrication based on UIP is illustrated in Figure 1. The ultrasonic impact tool converts the input electrical energy into mechanical energy through the internal ultrasonic transducer, which is connected to an ultrasonic horn and impact ball. The impact ball with ultrasonic mechanical vibration feeds vertically to workpiece surface. Under the action of ultrasonic mechanical vibration, the surface of stainless steel workpiece produces severe elastoplastic deformation, which results into the formation of point-like texture. Furthermore, in combination with the use of a motion control system to precisely control the trajectory of the workpiece, it is expected that complex-shaped textures such as microgrooves can be formed on stainless steel surface.

Although UIP method belongs to the category of mechanical processing technology, its processing mechanism is significantly different from the cutting method due to the absence of material removal. Specifically, workpiece material only undergoes elastic deformation and plastic deformation in UIP, which means that the surface texture is formed mainly by the plastic flow of the material. Theoretically, the sizes of the single texture fabricated by UIP have the following geometric relationship:

(*r* − *h*)^2^ + (*w*/2)^2^ = *r*^2^ (1)

where *r* represents the radius of impact ball, *h* and *w* represents the depth and width of the single texture fabricated, respectively. It can be deduced from the geometric relationship that the texture fabricated by UIP is a kind of shallow texture with a small ratio of depth to width. Since the time interval of each single impact is at the level of 10^−5^ s, successive ultrasonic impact events flatten the bottom surface of the texture, so the surface of the texture fabricated by UIP is smooth in theory. The detailed analysis can also be found in elsewhere [35].

## 3. Design of UIP-Based Device for Surface Texture Fabrication

### 3.1. Configuration of the UIP-Based Device

**Figure 2:**
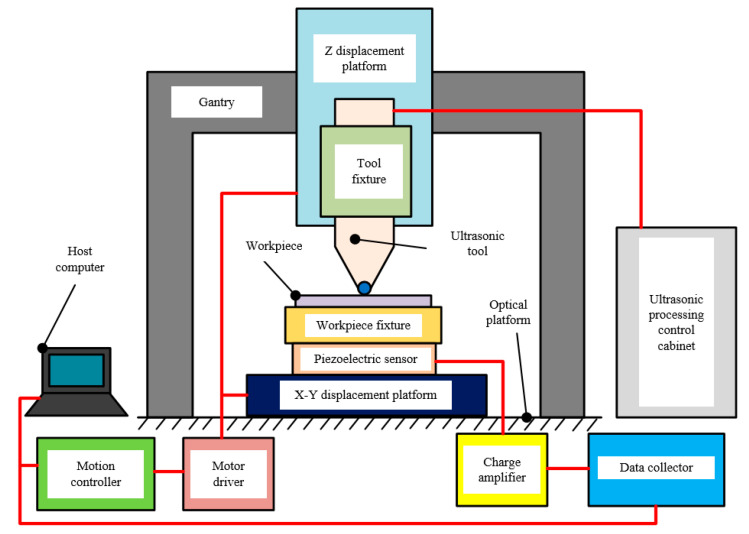
Configuration of the proposed UIP-based device.

According to the application requirements of ultrasonic impact machining, the overall scheme of the UIP device is designed, which controls parts of tool setting, force detection, precision motion control of workpiece and ultrasonic tool. The configuration of the as-proposed UIP device is illustrated in Figure 2, which consists of UIP system, motion control system, force detection system, and other mechanical structures. The UIP system is used for the conversion of electrical energy to mechanical energy, and is used to realize ultrasonic impact processing. The function of motion control system is to realize precise displacement control of the ultrasonic tool and workpiece. The force detection system is used to achieve accurate tool setting and monitor the stability of impact force during processing. The mechanical structures of the UIP device are used for connecting and fixing parts.

### 3.2. Functional Systems of the UIP Device

#### 3.2.1. Ultrasonic Impact Peening System

To realize the above-mentioned method of ultrasonic impact machining, a suitable UIP system is required. We chose a mature commercial ultrasonic impact surface modification equipment HK30C (Huawin Electrical & Mechanical Technology Co., Ltd., Shandong, China) to explore the application of the UIP method in surface texture fabrication. The UIP system mainly consists of an ultrasonic impact tool and an ultrasonic processing control cabinet. The ultrasonic generator in the ultrasonic processing control cabinet can produce sine wave electric signal with ultrasonic frequency. In addition, the control cabinet can also realize the circulating supply of lubricating fluid required during the UIP processing. 

**Figure 3:**
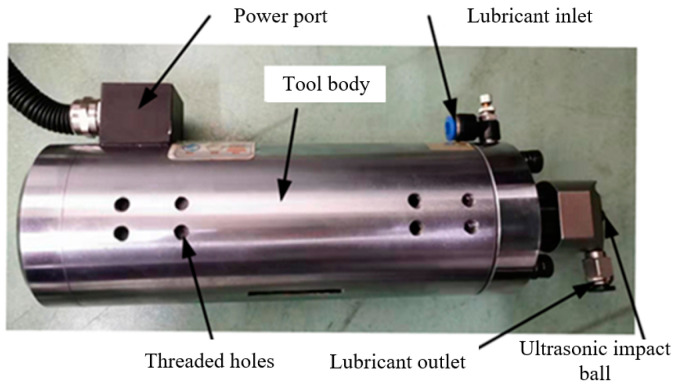
Ultrasonic impact tool.

Figure 3 shows the configuration of ultrasonic impact tool, the main features of which are: the tool body is implemented with transducer and horn inside; the power port provides ultrasonic electrical signal and feedback signal; the threaded holes are set for the fixing tool body; the ultrasonic impact ball is made of YG6 tool steel and can be changed in different diameters; the lubricant inlet and outlet are used for cooling and lubricating during processing. 

#### 3.2.2. Motion Control System

The UIP-based surface texture fabrication requires precise control of the displacement between tool and workpiece, so a motion control system needs to be designed. The motion control system mainly consists of a multi-axes motion controller, a Z displacement platform, and a X-Y two-dimensional displacement platform. An IMAC-FX motion controller from Delta tau is employed, which is cost-effective, high-performance, and can support up to four-axis motion control. WN203WA and WN200TA (Winner Optical Instruments Co., Ltd., Beijing, China) is employed for the two-dimensional X-Y displacement platform and the one-dimensional Z displacement platform, respectively. Specifically, the Z displacement platform is used for the vertical displacement feed of the ultrasonic impact tool. The X-Y two-dimensional displacement platform enables the workpiece to move in specific trajectory according to the pre-designed shape of surface texture. 

#### 3.2.3. Force Detection System

A force detection system is added to enable the detection of the contact between the tool and the workpiece surface. Meanwhile, it can more intuitively obtain the information of the impact force on the workpiece during UIP processing. The force detection system mainly consists of piezoelectric sensor, charge amplifier, and data collector. LC0511, LC0602-3, LC1608S (Lance Measurement Technologies Co., Ltd., Qinhuangdao, China) are used for piezoelectric sensor, charge amplifier, and data collector, respectively, which can collect both tool setting signal and ultrasonic frequency force signal.

### 3.3. Mechanical Structures of UIP Device

#### 3.3.1. Overall Layout of Mechanical Structures

**Figure 4:**
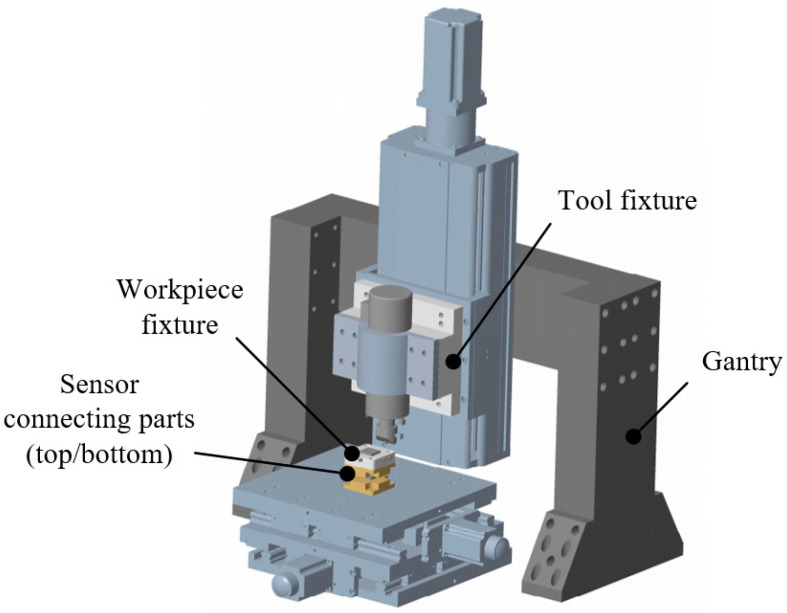
Mechanical structures of the proposed UIP-based device.

Figure 4 presents the overall layout of main mechanical structures of the UIP device, which mainly includes gantry, tool fixture, workpiece fixture, and sensor connecting parts. The mechanical structures are needed to support the entire UIP device and connect various functional components. Due to the unique shape and mechanism of the ultrasonic impact tool, and the special working condition of the workpiece under ultrasonic impact, the designs of the tool fixture and the workpiece fixture are emphasized. 

#### 3.3.2. Tool Fixture

**Figure 5:**
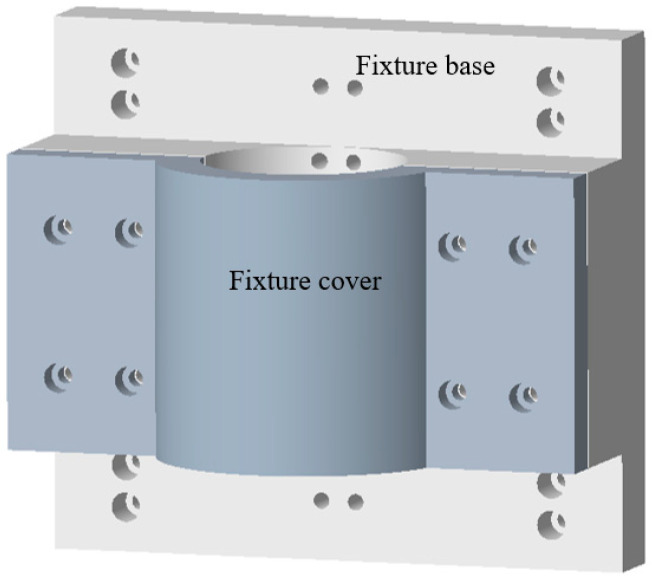
The structure of the tool fixture.

The tool fixture is specially designed according to the geometrical characteristics of the ultrasonic impact tool, which consists of a fixture cover, a fixture base, and connecting parts, as shown in Figure 5. The main assembly relations of the tool fixture are as follows: the fixture is fixed on the Z displacement platform by screws on both sides of the fixture base. The fixture and the ultrasonic impact tool are connected by screws in the middle of the fixture base, and the axis of ultrasonic impact tool is perpendicular to the X-Y two-dimensional displacement platform, so that the tool always impacts the workpiece surface vertically during processing. The fixture cover is screwed to the fixture base through countersunk holes on both sides, which simultaneously provides sufficient pressing force for the tool body. Moreover, the width dimension setting of the fixture cover should not interfere with the power port of the tool. The semicircle dimensions of the fixture cover and base are equal to the radius of the tool body with appropriate tolerances. The two-part design facilitates the assembly of the ultrasonic tool and ensures the positioning of the tool. 

#### 3.3.3. Workpiece Fixture

**Figure 6:**
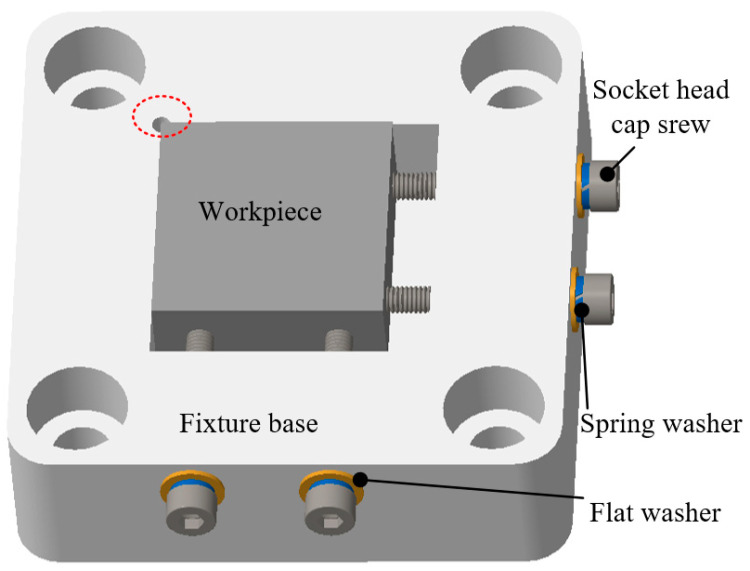
The structure of the workpiece fixture.

The selected workpiece has a size of 20 × 20 × 10 mm. While the workpiece is thin and difficult to be clamped under the ultrasonic impact, multiple degrees of freedom need to be fixed to ensure the machining accuracy and stability. Ordinary fixture has the problem of suspending fixed workpiece or easily colliding with the tool, so the fixture is especially designed according to the experimental scene of UIP. The workpiece fixture consists of a fixture base, socket cap head screws, spring washers, and flat washers, as shown in Figure 6. The main assembly relations of tool fixture are as follows: a placement cavity is set in the middle of the fixture base. One of the corners of the square cavity is machined with an avoidance hole, which can avoid positional interference between the milling fillet of the square cavity and the corner of the square workpiece. The depth dimension of the placement cavity is slightly smaller than the height dimension of the workpiece used in experiment, to ensure that the workpiece can protrude a certain distance from the surface of workpiece fixture. In such a way, accidental collision between the ultrasonic impact tool and the workpiece fixture can be prevented during processing. Two threaded holes are respectively set on the front and right sides of the fixture base. Then, socket head cap screws, spring washers, and flat washers are used to tightly fix the workpiece to prevent the workpiece from being misplaced during processing. In addition, four corners of the fixture base are respectively provided with a countersunk hole, which can be used for screw connection with the top sensor connecting part. It should be mentioned that the bottom surface of the screw used for fixing needs to be processed smoothly.

**Figure 7:**
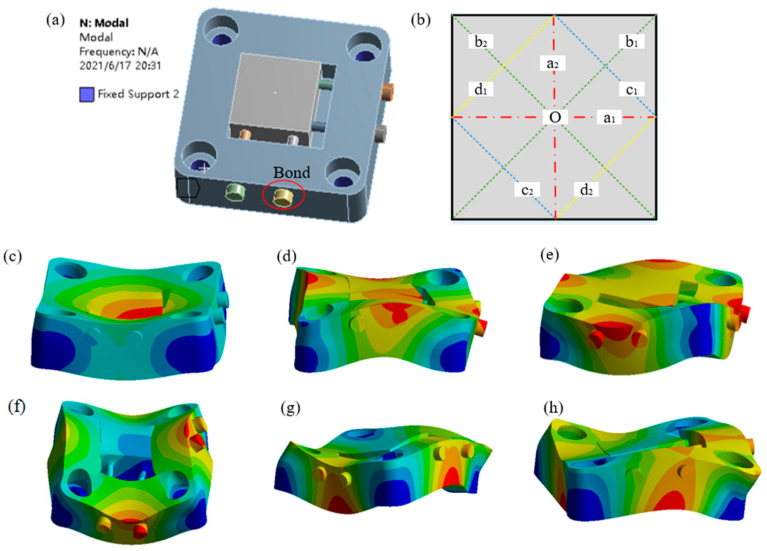
Mode analysis of the workpiece fixture. (**a**) Constraint; (**b**) Schematic diagram of indicator line; (**c**–**h**) Mode 1−6.

The workpiece fixture is the structure mostly affected by the ultrasonic impact force, which may affect its structural stability and machining accuracy. In particular, finite element analysis is carried out on the structure of the workpiece fixture. As shown in Figure 7a, the workpiece fixture model is simplified, and the fasteners between the screw head and the workpiece fixture base are replaced with “Bond” contact. The countersunk holes at four corners of the fixture base are set as “Fixed support”. The first six-order vibration mode of the workpiece fixture is shown in Figure 7c−h. In mode 1, the fixture swings up, and down in the central area. In mode 2, the fixture twists along *b*_2_ line. In mode 3, the fixture twists along *b*_1_ line. In mode 4, the fixture bends along *a*_1_ line and *a*_2_ line at the same time. In mode 5, the fixture twists along *c*_1_ line and *c*_2_ line at the same time. In mode 6, the fixture twists along *d*_1_ line and *d*_2_ line at the same time. In addition, the natural frequencies of the first six-order mode are shown in Table 1.

**Figure 8:**
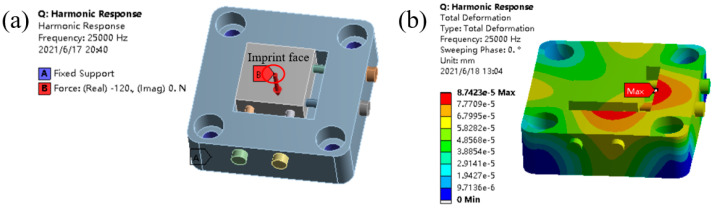
Harmonic response analysis of the workpiece fixture. (**a**) Load and constraint; (**b**) Deformation.

It can be found that the ultrasonic processing frequency (25,000 Hz) is close to the natural frequency of the fourth-order mode, which may cause the workpiece fixture to resonate during UIP processing and subsequently affect machining accuracy. Therefore, harmonic response analysis of the workpiece fixture is also carried out by finite element analysis. As shown in Figure 8a, based on the constraint of the modal analysis, the “Imprint Face” with a diameter of 1 mm on the workpiece surface is applied a “Force” with a magnitude of 120 N and a frequency of 25,000 Hz. As shown in Figure 8b, it can be seen that under the action of ultrasonic impact, the maximum deformation appears at the upper edge area of the placement cavity, and the maximum deformation is 0.09 μm, which is very small to ensure the requirement of high-precision processing. It should be mentioned that the above “Force” parameters come from the data obtained by the force detection system under appropriate processing parameters. The simulation results verify the rationality of the initial structure design of the workpiece fixture. 

## 4. Surface Texture Fabrication Experiment by UIP

According to the design scheme of the functional systems and mechanical structures, a UIP-based device is built for the fabrication of surface texture on stainless steel. Before each UIP processing, the vertical displacement of the impact ball is adjusted to enable it close to the surface of the workpiece. When the piezoelectric sensor starts to detect the change of the impacting force, the tool setting is realized. In addition, according to the processing depth requirement of the texture, a certain amount of pre-compression feed is provided. During each UIP processing, the force detection system monitors the information of the force, which can be used to judge the uniformity and stability of the ultrasonic impact force to ensure machining accuracy. 

**Figure 9:**
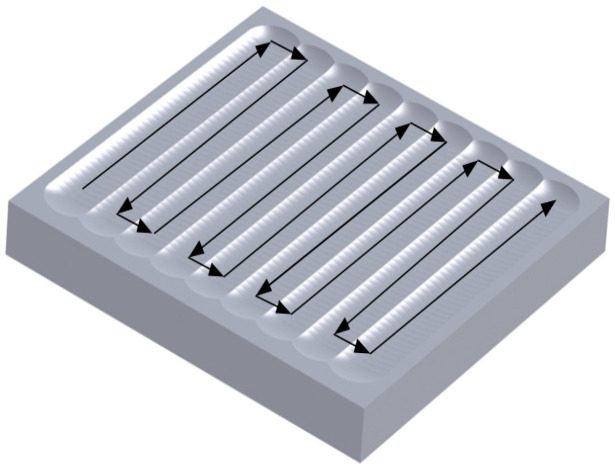
Schematic diagram of UIP processing trajectory.

The used workpiece size is 20 × 20 × 10 mm in the experiment. 316L cold-rolled austenitic stainless steel is selected as the workpiece material for this experiment. The workpiece has an average grain size of 15 μm, a young’s modulus of 192 GPa and a yield strength of 300 MPa. After polishing, the surface roughness and flatness of the workpiece are 100 nm and 1 μm, respectively. Figure 9 shows the trajectory of the workpiece during UIP processing. Table 2 lists the basic processing parameters used in the UIP experiments.

**Figure 10:**
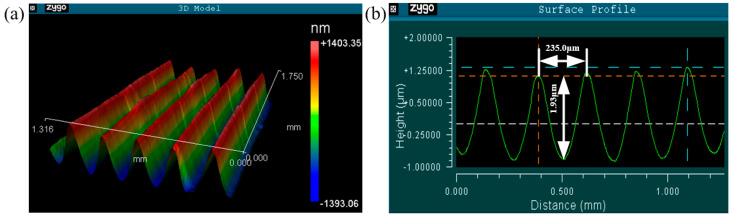
The morphology of fabricated array microgroove. (**a**) 3D contour; (**b**) Section profile.

The morphology of the fabricated surface microgrooves is characterized by ZYGO NewView 6300 white light interferometer. The detection data of sampling areas is shown in Table 3. Figure 10 shows the microscopic morphology of the test area No. 2. It can be found that the texture morphology is regular and has good precision and consistency: its width periodicity is 240 μm and the deviation is −5 to 0.8 μm; its depth is 2 μm, the deviation is −0.20 to 0.14 μm. Thus, it can be found that UIP has an excellent advantage in the manufacturing of shallow surface textures with a very low aspect ratio on stainless steel. The detailed characterization of surface morphology of as-fabricated aligned microgrooves can also be found in recent work [35].

**Figure 11:**
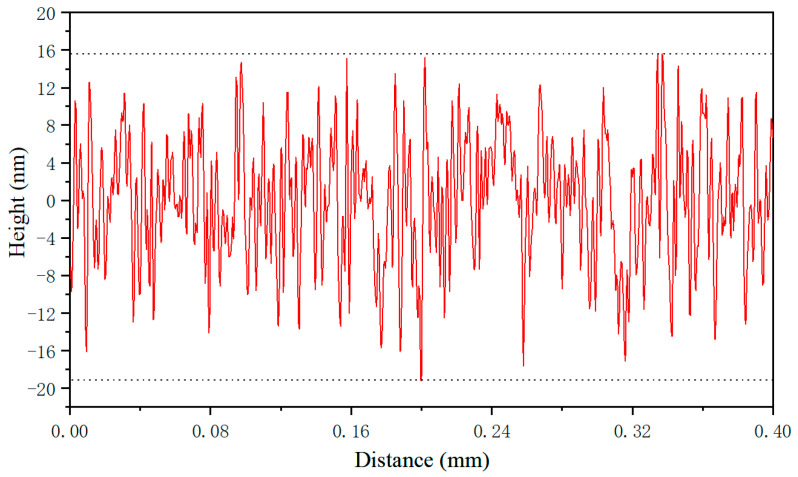
The roughness of microgroove bottom surface.

The surface roughness of the bottom of the microgroove is measured by the Mitutoyo SJ210 surface roughness meter. The measurements are taken along the length of the microgroove, with a sampling length of 0.08 mm, a detection length of 0.4 mm and a detection speed of 0.25 mm/s. Figure 11 plots the variation of measured surface roughness of the microgroove surface with distance. It can be seen from Figure 11 that the maximum height of profile (*R_z_*) is 34 nm, the arithmetical deviation of the profile (*R_a_*) is 5 nm, indicating that the surface of microstructure fabricated by UIP is very smooth. The detailed characterization of surface roughness of microgroove can also be found in recent work [35]. The textured surface combines the smoothing effect of UIP originally used for surface treatment, which is related to the mechanism of the UIP method: under the high-frequency and high-energy action of ultrasonic impact, the grains of the surface layer at a certain depth are broken and refined, the structure is strengthened and receives new distribution, and the surface produces uniform plastic deformation. Furthermore, the time interval between adjacent impacts is on the order of 10^−5^ s. 

Therefore, the UIP exhibits significant advantages in the manufacturing of high-precision surface textures on stainless steel over both LST and diamond cutting. Specifically, the depth of textures by UIP can easily go down to a few micrometers, which is quite challenging for LST. Furthermore, the textures by UIP possess larger width-to-depth ratio than that by LST. In addition, compared with the textures by the diamond cutting, the textures by UIP possess similar precision but significantly lower cost due to the use of carbide tool rather than diamond tool.

## 5. Conclusions

In this subject, a UIP device that realizes the fabrication of complex-shaped texture is designed based on the analytical analysis of UIP principle. The proposed UIP device consists of an UIP system, a motion control system, a force detection system and other mechanical structures. This device is applied to fabricate array microgrooves on 316L stainless steel surface under the processing conditions of a frequency of 25 kHz, an amplitude of 6 μm, a feed speed of 2 m/min, a processing depth of 6 μm and a width period of 240 μm. The fabricated array microgrooves indicate a high precision. Specifically, while the width periodicity is 240 μm and the depth is 2 μm, the corresponding maximum deviation in width and depth is 2.08% and 10% along entire groove, respectively. In addition, the *R_z_* and *R_a_* of bottom of the fabricated groove is 28.7 nm and 5 nm, respectively. Thus, current work preliminarily demonstrates the feasibility of applying UIP in the manufacturing of high-precision surface texture on stainless steel with low cost.

## Figures and Tables

**Table 1:** The natural frequencies of the first six-order mode.

Mode Order	1	2	3	4	5	6
**Frequency (Hz)**	16,779	23,367	23,631	24,942	25,907	25,929

**Table 2:** Parameters of UIP experiment.

Parameter	Value
Frequency (kHz)	25
Amplitude (μm)	6
Feed speed of workpiece (m/min)	2
Feed depth of tool (μm)	6
Width periodicity of array microgroove (μm)	240
Diameter of impact ball (mm)	6

**Table 3:** The width periodicity and depth of fabricated array microgroove.

Sampling Area NO.	Width Periodicity (μm)	Depth (μm)
1	240.7	2.10
2	235.0	1.93
3	240.8	1.80
4	240.0	2.12
5	239.7	2.14
6	236.8	2.08
